# Post-stroke pathway analysis and link with one year sequelae in a French cohort of stroke patients: the PAPASePA protocol study

**DOI:** 10.1186/s12913-019-4522-2

**Published:** 2019-10-29

**Authors:** S. Broussy, F. Rouanet, E. Lesaine, S. Domecq, M. Kret, M. Maugeais, F. Aly, P. Dehail, A. Bénard, J. Wittwer, R. Salamon, I. Sibon, F. Saillour-Glenisson

**Affiliations:** 1 Université de Bordeaux, Institut de Santé Publique d’Epidémiologie et de Développement, Centre INSERM U1219 Bordeaux Population Health center, 146 rue Léo Saignat, 33076 Bordeaux Cedex, France; 2 INSERM, ISPED, Centre INSERM U1219-Bordeaux Population Health, F-33000 Bordeaux, France; 3 Pôle des Neurosciences Cliniques (I.S., F.R.) CHU Bordeaux, Bordeaux, France; 4 CHU de Bordeaux, Pôle de santé publique, Service d’Information Médicale, F-33000 Bordeaux, France; 5 Physical and Rehabilitation Medicine Unit, EA4136, Bordeaux University Hospital, University of Bordeaux, Bordeaux, France; 6 Neurology, Stroke Unit, INCIA CNRS UMR 5287, Bordeaux University Hospital, University of Bordeaux, Bordeaux, France

**Keywords:** Stroke, Pathways, Sequelae, Health services research

## Abstract

**Background:**

Stroke is a health problem with serious consequences, both in terms of mortality, and after-effects affecting patient quality of life. Stroke requires both urgent and chronic management involving the entire health care system. Although large variability in the management of stroke patients have been noticed, knowledge of the diversity and the scalability of post-stroke pathways, whether it is the care pathway or the life pathway, is currently not sufficient. Moreover the link between post-stroke pathways and patients sequelae have not been yet clearly defined. All this information would be useful to better target the needs to improve stroke patient management.

The purposes are to identify the post-stroke life pathways components associated with sequelae (activity limitations – main purpose, cognitive disorders, anxio-depressive disorders, fatigue, participation restrictions) at 3 months and 1 year post-stroke, to define a typology of life pathways of patients during the post-stroke year and to analyze the social and geographical inequalities in the management of stroke.

**Methods:**

Design: a prospective multicenter comparative cohort study with a follow up to 1 year after the acute episode.

Participant centers: 13 hospitals in the Aquitaine region (France).

Study population: patients diagnosed with a confirmed ischemic or hemorrhagic stroke included in the Aquitaine Observatory of Stroke (ObA2) cohort and voluntary to participate.

Data sources are existing databases (ObA2 database and the French National Health Data System - SNDS) to collect information about care pathways, patient characteristics and stroke characteristics and Ad hoc surveys to collect information about life pathways and post-stroke sequelae. The endpoints of the study are post-stroke activity limitations evaluated by the modified Rankin score, other post-stroke sequelae (Cognitive disorders, anxio-depressive disorders, fatigue, restriction of participation) assessed by standardized and validated scales and Clusters of patients responding to pathways with common or similar characteristics.;

**Discussion:**

By integrating a longitudinal dimension and relying on a large cohort, the project will make it possible to identify the sources of disturbances and the factors favorable to the outcome of the life pathways, important for the planning of the offer and the management of the public policies concerning stroke pathways.

**Trial registration:**

ClinicalTrials.gov ID: NCT03865173, March 6th, 2019.

## Background

### Stroke: a worldwide public health issue

Stroke is the leading cause of acquired physical disability in adults worldwide. It represents a real public health issue in terms of frequency, severity and cost.

Worldwide burden of stroke is huge, with 10.3 million new strokes per year and 113 million disability-adjusted life years (DALYs) per year [1, 2]. Stroke is the second cause of death and the third cause of disability adjusted life years worldwide [3]. Every year in France, about 110,000 patients present with a stroke, responsible for about 30,000 deaths, and 500,000 persons live with various level of disability following a stroke [4]. Many lifestyle risk factors (high blood pressure, diabetes, smoking, poor nutrition, sedentary lifestyle, etc.) [2, 5] and an aging population contribute to a steady increase in the incidence and prevalence of stroke.

Consequently, the socioeconomic impact of stroke is considerable. The annual cost of stroke in Europe is estimated to be €45 billion: €20 billion for direct care, €9 billion related to loss of productivity and €16 billion for informal care [6]. In France, in 2007, the economic burden of stroke amounted to about 8.6 billion euros [7]. This burden goes beyond the expenses incurred for the initial event. The expenditure of care during the first year represents a third of the direct costs [8]. The impact in terms of indirect costs, loss of income for active patients, time spent by family caregivers, accommodation of dependent people, adds to the burden of this pathology.

### Stroke: a severe disease requiring both urgent and chronic management

It is the very nature of the pathology, but also the consequences that stroke entails, which requires a management, both, extremely urgent and chronic. Indeed, stroke is a medical emergency that requires care in the shortest time. The advents of stroke units, of thrombolysis [9–11] and more recently of thrombectomy [12] have revolutionized acute stroke care, playing a decisive role in terms of vital and functional prognosis. However, the increase of stroke survivors related to major improvements in acute management and the aging of the population leading to an increase in the needs for post-stroke facilities [13, 14].

### Post-stroke sequelae

The consequences of stroke are potentially dramatic, with, on the one hand, high mortality, and on the other hand, for those who survive, short-term, medium-term and long-term sequelae requiring particularly heavy daily care. Sequelae spectrum is wide. They are physical (motor disorders, aphasia, sensory disorders), psycho-intellectual (cognition, depression), functional (activity limitation), or socio-professional (driving, loss of work, family difficulties) sequelae [15]. The World Health Organization’s International Classification of Functioning, Disability and Health (WHO-ICF) [16] gives a conceptual framework that can aid classification of the sequelae in three categories: disabilities (the direct loss of function), limitations of activities (formerly called disability) and participation restrictions (called handicap).

Sequelae can be measured by validated and standardized scales [17–23]. The most commonly used and specific ones are the modified Rankin score (mRS) [21] or the Barthel index (BI) [20].The mRS measures the degree of dependence in activities of daily living. This tool is widely used to measure the functional impact in clinical trials. The BI is an ordinal scale used to measure performance in activities of daily living.

### Predictive factors and outcome

Many studies have analyzed the stroke functional predictive factors [24, 25]. They can be sorted into three categories: variables related to the patient’s previous condition (age, sex, patient history, antecedents and autonomy before stroke), variables related to stroke (severity of hemiplegia, type of stroke - cerebral infarction or intracerebral hemorrhage) and finally variables related to the patient’s life pathways (speed of care in the acute phase, access to a stroke unit, early return home, social and familial environment). These factors interact with each other.

Among the variables related to the patient’s previous state, it is established that the autonomy, assessed by the modified Rankin score or the Barthel index, prior to stroke and at the inaugural event, is decisive in term of functional outcome.

Among the variables related to stroke, the initial severity of stroke, measured by the Institutes of Health Stroke Scale (NIHSS) [26], is one of the variables most strongly associated with the functional outcome.

With regard to the elements of the care pathway, several studies have showed that short and long-term outcomes after stroke are associated with better acute care, such as thrombolysis and stroke unit care, as well as risk factor management before and after stroke. As regards post-stroke pathways and outcome, only the role of rehabilitation treatment after a stroke [27–30] and the early supported discharge [31–34] have been shown as linked to functional outcomes. Reeducation must be initiated in stroke units, according to the patient needs, and continued and coordinated by a multidisciplinary team. However, no information exists on the functional role of further temporal and spatial elements of the downstream pathways following stroke.

### The stroke life pathways

The efforts of professionals and healthcare system regulators to improve the management of stroke patients first focused on the acute phase of the stroke management [35], and in particular hospital care within stroke units.

On the ground, however, multiple barriers impede the quality, fluidity and overall the efficiency of stroke care pathways. These obstacles mainly concern downstream support. Difficulties of referral to adapted structures after the acute episode are often encountered, with, as a consequence, number of patients admitted to rehabilitation remaining below public health objectives [36]. This represents a real loss of opportunity for some patients, impairing the speed of the implementation of rehabilitation which is a key element of functional outcome after a stroke. In addition, access to prevention, specialized care and medico-social structures is limited. The impact of these defects of care on functional outcomes have never been measured. Face to this lack of knowledge, it is difficult to define post stroke strategy to improve patient health. Furthermore, even more than the need for care, patient management after a stroke involves examining the situation of the person as a whole: his state of health, his autonomy in the daily life, his family and social environment and his resources. These different dimensions will have to evolve throughout pathway and will require a regular adjustment of the mods of accompanying.

Usually, three categories of management pathways for patients with chronic disease are identified: care pathways, health pathways and life pathways [37].

The care pathway is based on the right coordination of the various professional interventions related directly or indirectly to care. It integrates ambulatory and inpatient care (primary care and hospitalization, home hospitalization, follow-up and rehabilitation care, long-term care unit). The main objective of care coordination along the care pathway is to ensure that care is provided, to avoid duplication of unnecessary tests and to increase the safety and efficiency of care through reliable and shared information in real time [37].

Health pathways meet patient prevention, medico-social and social needs. These integrate the care pathways articulated with, on the one hand, preventive actions and, on the other hand, medico-social and social support, and the home return home (medico-social establishments, temporary accommodation and respite structures, home services, etc.) [37].

Finally, the life pathways refers to a global accompaniment of the person, based on the examination of his situation as a whole: personal life project, autonomy in daily life, state of health, family and social environment, resources, etc. These different dimensions are subject to change and require regular adjustment of the support arrangements implemented, involving multiple stakeholders and coordinated actions by the stakeholders [37, 38].

Pathways of stroke patients are not linear, but are divided into management sequences within health or medico-social facilities, evolving according to the complications and events directly or indirectly related to the stroke. Stroke patient care requires good organization and articulation of the different links in the care chain and the different care actors and, must consider the consequences of stroke on the person’s situation in his or her life context. Thus, the notion of “life pathways” is preferable to these of “care pathways” or “health pathways” [37, 38]. It is all the parameters integrated in the life pathways that make the post-stroke life pathways so complex that they are so far poorly known and poorly identified. Knowledge on their components, their typologies and on the functional role of their components is needed to construct a strategy taking into account post-stroke health as a whole.

### The French stroke management policy

In France, the 2010–2014 National Stroke Action Plan was launched to reduce the frequency and severity of stroke-related sequelae by improving the organization of regional care chain for stroke management. A reference stroke care pathway has been defined since the emergency to the relay with the medico-social actors and structures and / or the return home [39–41]. It defines the offer of care to be mobilized, for a standard stroke patient, as part of a linear pathway. The program of Health System Modernization [42] aims to develop and implement a patient integrated care pathway with a perspective of outcome.

Consequently, knowledge on the link between downstream pathway and functional outcomes will help constructing this program.

In the Aquitaine region, south western of France, the Aquitaine Stroke Observatory, ObA2, was set up by the regional health agency (ARS), as part of the regional plan for the fight against cardio-neurovascular diseases. This is a regional cohort of patients with a recent stroke, diagnosed and managed in the short-stay services of the hospitals in Aquitaine hosting more than 30 strokes per year. ObA2 objectives are: to 1) describe the management of stroke (practices, delays, referral of patients, etc.) prior to hospitalization, during hospitalization and in the post-hospital phase, 2) describe the population of stroke patients in socio-demographic and clinical terms, 3) monitor the population of these patients in terms of occurrence of complications during the stay, morbi-cardio-neuro-vascular mortality at 1 year and disability at 1 year. ObA2 is the only stroke cohort in France covering a whole region, allowing the inclusion of a large and heterogenic panel of stroke patients. The Observatory is a tool for improving practices and care pathways for patients with stroke. It is also used to develop research projects around stroke management. It is a tool to provide information to decision-makers to build a regional health strategy on the organization and care chain to help them structure the care pathways.

The PAPASéPA (Parcours des Patients victimes d’accident vasculaire cérébral et Séquelles Post-Accident vasculaire cérébral) project was built to address the lack of information on the components of the downstream life course and the link between these components and sequelae. In the ObA2 cohort dynamic, detailed data on the components of the life pathways, on the link between life pathway and sequelae could be used to better identify the needs to improve the health care system. We are in a global perspective of analysis of the life pathway of stroke patients, pre-hospital, hospital and post-hospital phases, and this for 1 year after acute care.

## Objectives

The main objective of the PAPASéPA project is to identify the life pathways components of stroke patients associated with the activity limitations measured by the Modified Ranking Score (mRS), at 3 months and 1 year after the acute episode.

The secondary objectives are to: 1) identify the life pathways components of stroke patients associated with other types of sequelae (cognitive disorders, anxio-depressive disorders, fatigue, restriction of participation) at 3 months and 1 year after the acute episode; 2) define a typology of the life pathways of stroke patients in Aquitaine, from acute care to 1 year of follow-up and 3) analyze the social and geographical inequalities in the management of stroke in Aquitaine.

## Methods/design

### Study design

**Fig. 1:**
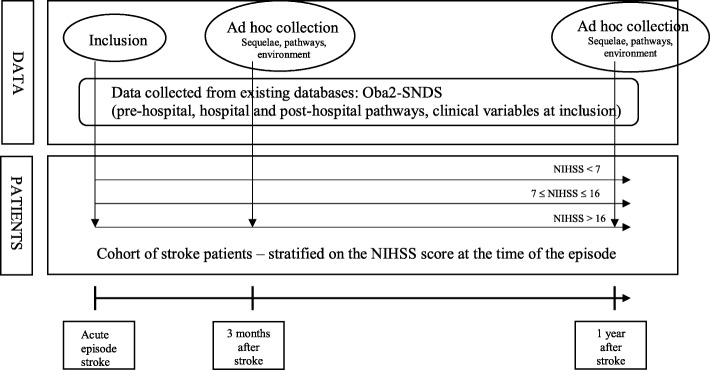
Structuring and design of the PAPASéPA project

The study design is an observational prospective cohort of stroke patients included in ObA2 with a patient follow up period for 1 year following stroke. Continuous data collection of care pathways will be carried out from stroke throughout the year of follow-up from existing databases (the ObA2 data base and the French National Health Data System (*Système National des Données de Santé -* SNDS); moreover, ad hoc collection of sequelae, pathways and socio-economic and environmental data is planned at three times (initial episode, at 3 months and 1 year after initial episode) (Fig. 1). The cohort is divided into three strata based on the severity of the patient’s initial clinical condition, as measured by the NIHSS score (≤6, [7–16], > 16) at the end of the hospital stay (Fig. 1).

**Fig. 2:**
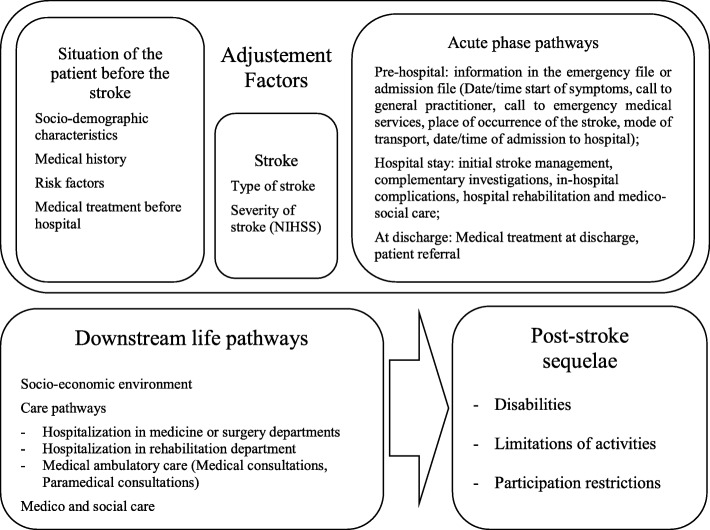
Causal model of the link between downstream pathway and post-stroke sequelae

Our analysis is based on the causal model linking life post-stroke pathway components and post stroke sequelae (Fig. 2). The adjustment factors are patients or stroke characteristics or stroke acute pathway elements/components identified in the literature as associated with stroke functional outcomes.

### Population and study sample

#### Study population

The study population involves patients diagnosed with a confirmed ischemic or hemorrhagic stroke included in the ObA2 cohort and managed in one of the 13 hospitals of the Aquitaine region, south Western France participating to Oba2. These 13 hospitals include 5 stroke units, 2 hospitals with neurologist and 6 hospitals without a neurologist.

#### Patient selection criteria

The inclusion criteria into the PAPASéPA cohort are: inclusion criteria to ObA2 (being over 18 years of age living in metropolitan France, suffering from a recent stroke whose diagnosis was confirmed by a neuro-vascular physician) and as additional criteria, Giving consent to participate to PAPASéPA).

Patients will be identified prospectively as they are included in ObA2. The inclusions will be carried out continuously and prospectively from the date of the beginning of inclusions (planned to be in september 2019) until the required number of subjects is obtained in each strata.

### Data sources and data collection

#### Data sources

Three types of data sources are considered (Table 1):

**Table 1:** Summary of Stroke Patient Data Collection – PAPASéPA

	Inclusion visit	Follow-up at 3 months post-stroke	Follow-up at 1 year post-stroke
Verification of eligibility criteria	✓		
Patient information^1^ + Oral consent	✓		
Data on the care, socio-demographic and clinical pathways (history, severity, complication during the stay ...) to the acute phase (pre-hospitalization and hospitalization) from the ObA2 Observatory ^2^	✓		
Hospitalization data, consultations, acts, outpatient care from the SNDS^3^	Continuous collection		
Clinical data (ad hoc collection):			
- Severity of stroke: NIHSS at discharge	✓		
- Risk factors (alcohol)	✓		
Stroke pathways data (ad hoc collection)^4^			
- Patient referral ^4^	✓		
- Level of education ^5^	✓		
- Professionnal activity ^5^	✓		
- Marital status ^5^	✓	✓	✓
- Cohabitation status ^5^	✓	✓	✓
- Ability to drive ^5^	✓	✓	✓
- Place of life ^5^	✓	✓	✓
- Participation in a therapeutic education program ^5^		✓	✓
- Medical and social care ^5^		✓	✓
- Allowances for disability ^5^		✓	✓
- Family/Caregiver ^5^		✓	✓
- Development and technique to improve autonomy and home care ^5^		✓	✓
- Technical assistance for walking ^5^		✓	✓
- Legal background ^5^		✓	✓
Sequelae post-stroke (ad hoc collection)			
Cognitive disorders (TICS*) ^5^		✓	✓
Anxiety-depressive disorders (HADS**)(Self-assessment)^6^		✓	✓
Fatigue (FSS***) (Self-assessment)^6^		✓	✓
Activity limitations (BI ǂ) (Self-assessment)^6^	✓	✓	✓
Activity limitations (MRSǁ) ^5^	✓	✓	✓
Participation restriction (CIQ-R§) (Self-assessment) ^6^		✓	✓

*Telephone Interview for Cognitive Status; **hospital anxiety and depression scale; ***Fatigue Severity Scale; ǂBarthel Index; ǁmodified Ranking scale; §Community Questionnaire

^1^ Address; telephone number

^2^ Type of stroke; Severity (NIHSS) at admission; Gender; Age; History of Neuro-cardiovascular; History of neuropsychiatric disorders; Anticoagulant / antiaggregant treatments; Rankin before stroke; Date/time start of symptoms; Date/ time call to general practitioner; Date/time call to emergency medical services; Place of occurrence of the stroke; Mode of transport; Date/time of admission to hospital; Type of hospitalization unit; Performing thrombolysis / tele-thrombolysis / thrombectomy; Type of antithrombotic treatment; Complication during stay; duration of stay

^3^ Number / type / duration of hospitalization in medicine or surgery, rehabilitation, psychiatry departments. Hospitalization at home; Number / type of medical and paramedical consultations at hospital; Medical ambulatory care: number / type of medical consultations (general practitioner, neurologist, functional rehabilitation physician, cardiologist) and number of care periods, total duration of each care period / type of paramedical consultations (physiotherapy; speech therapy; orthopsy); Quantity of drug deliveries (antiaggregant, antihypertensive, statin or anticoagulant treatment, analgesic), external medical consultations for neurology, rehabilitation, cardiology in health institutions; Vital status of patients

^4^ Patient referral (admission to the hospitalization area of the institution, transfer to another institution), Level of education (INSEE classification); Marital status (Married - Accessed - Partner; Separated - Divorced – Widowed; Single); Cohabitation status (couple; in family- friends; institution), Ability to drive (yes, no); Place of life (home, institution, medico-social structure), Medical-social care (services to support social life, services to help and support at home, services to provide nursing care at home, services to provide medical and social support), Family/Caregiver (yes, no); Development and technique to improve autonomy (yes, no); Legal background (Curatorship, Guardianship)

^5^ Data collected by telephone by the nurse

^6^ Postal delivery to the participant’s home

Extraction of data from two existing databases: the ObA2 cohort and the French National Health Data System (*Système National des Données de Santé* – SNDS). The extractions from existing databases will provide the main information about care pathways, patient characteristics and stroke characteristics.

The extraction from the ObA2 database will provide information about patient socio-demographic characteristics (date of birth, sex, place of residence), clinical (medical history, risk factors, medical treatment before hospital, type of stroke) and acute phase pathways (pre-hospital: date/time start of symptoms, date/time call to general practitioner, date/time call to emergency medical services, place of occurrence of the stroke, mode of transport, date/time of admission to hospital; hospital stay: initial stroke management, complementary investigations, in-hospital complications, hospital rehabilitation and medico-social care; at discharge: NIHSS, medical treatment at discharge, patient referral) (Table 1).

The National Health Data System regroups healthcare reimbursement data from in-patient and outpatient care, as well as causes of death. It provides data on claims paid for each patient by the French National Social Security System and is therefore the main source of information on healthcare costs. This database contains patient data (age, sex, place of living, long-term and chronic diseases, date of birth, date of death, health insurance scheme, benefit of free complementary insurance for lower-income people), all consultations and visits to General Practitioners (GPs) and ambulatory care specialists (but nothing about their content), all medical technical procedures, all dispensed medical devices and drugs, all lab and diagnostics tests but not their results, and providers’ level data (their activity and sales turnover, geography, prescribing behaviour). In addition, mortality and cause of death data are also available in this database.

Extractions from the SNDS will cover the time period from September 2019 to August 2020 and provide information about the post-stroke care pathway (Table 1):

- ◦ Number / type / duration of hospitalization in medicine or surgery departments, rehabilitation department, psychiatry department and hospitalization at home. Hospitalisations will be classified into three categories corresponding to hospital stays with different clinical meanings with regard to the evolution of stroke (readmission for management of stroke recurrence and post-stroke complications, readjustment for post-stroke follow-up and secondary prevention of stroke and re-hospitalizations to manage a clinical situation not related to stroke);
- ◦ Number / type of medical and paramedical consultations at hospital;
- ◦ Medical ambulatory care: number / type of medical consultations (general practitioner, neurologist, functional rehabilitation physician, cardiologist) and of paramedical consultations (physiotherapy, speech therapy, orthopsy), number of care periods, total duration of each care period;
- ◦ Quantity of drug deliveries (antiaggregant, antihypertensive, statin or anticoagulant treatment, analgesic);
- ◦ Vital status of patients.

**Ad hoc collection** at any of three points in the study (initial episode, at 3 months and 1 year after the initial episode).

The collection time for each variable is specified in Table 1. The categories of variables collected are as follows:

- ◦ **Clinical data:** risk factors (Alcohol), severity of stroke at discharge (NIHSS)
- ◦ **Stroke life pathways data:** patient referral, level of education, marital status, cohabitation status, professional activity, ability to drive, place of life, participation to a therapeutic education program, medical and social care, allowances for disability, presence of family and caregiver, development and technique to improve autonomy and home care, technical assistance for walking, legal background.
- ◦ **Post-stroke sequelae assessment**

A selection of scales measuring each of the dimensions explored in our project was selected, based on elements of feasibility, frequency of use in the exploration of the dimension considered and metrological performance (validity and reproducibility): The mode of collection of sequelae has been adapted according to the validated mode of administration for each of the scales. The following sequelae are assessed:

- **Cog impairment** using the **French Telephone Interview for Cognitive Status Modified (F-TICS-m).** The TICS scale is a tool for screening for memory disorders. It evaluates time orientation, spatial orientation, immediate memory, calculation, semantic memory, delayed recall, language, understanding of an instruction. Cut offs of 24 and 34 are used (a score less than 24 corresponds to dementia, a score between 24 and 33 corresponds to mild cognitive impairment and a score more than 34 corresponds to a normal condition). This scale was validated in French by telephone [19].
- **Depression and anxiety** using the **hospital anxiety and depression scale (HADS)**. The anxiety and depression score through the HADS seeks to identify an anxiety-depressive symptomatology and assess its severity. It is composed of 14 items divided into 2 scores of seven items each: a depression score (one for dysphoria, one for deceleration and five for the anhedonic dimension) and an anxiety score. Each item is rated from 0 to 3, allowing to obtain 2 scores. The maximum score for each score is 21. This scale was validated in French by telephone and in a self-administered collection mode [20].
- **Fatigue** using the **Fatigue Severity Scale (FSS).** The fatigue score through the FSS measures the impact of fatigue on different functions. This is a short questionnaire of nine questions, to which the patient answers on a scale from 1 to 7. The overall score is the average of all the scores obtained on each of the 9 questions. The higher the result, the greater the fatigue. A threshold of 5.5 is often used to differentiate high and low fatigue. This scale was validated in French in a self-administered collection mode [21].
- **Genito-sexual disorders** using closed-ended question “yes/no”.
- **Limitations of activities** using the **modified Rankin Scale (mRS)** [23] and the **Barthel Index (BI)** [22]. The modified Rankin Scale (mRS) and the Barthel Index (BI) are commonly used scales measuring the actual abilities of the stroke patient in the basic activities that determine autonomy. The mRS is a more global scale, which measures functional independence rather than the performance of specific tasks. In this way, mental as well as physical adaptations following neurological deficits are assessed. The scale consists of 7 levels, from 0 to 6, with 0 corresponding to no symptoms and 6 corresponding to death. Cut offs of 2 is used (a score less than or equal to 2 corresponds to independence, a score greater than 2 corresponds to dependence). This scale was validated in French by telephone and in self or hetero-administered data collection mode. The IB was developed in 1965 [22] and later modified by Granger and colleagues [43] to measure patient performance in 10 activities of daily living. The IB is considered a sensitive and reliable disability scale for stroke patients. The IB items can be divided into two groups: one, related to self-care (diet, grooming, bathing, clothing, urinary and anal continence, and toilet use) and the other, related to mobility (ambulation, transfers and stair climbing). The maximum score is 100, which indicates that the patient is totally independent in physical functioning. The lowest score is 0, which represents a totally dependent bedridden state. A dependency to consider a return home is usually defined by a score greater than or equal to 60. The IB is a scale validated in French in a data collection mode by interview (hetero-administered).
- **Participation restrictions** using the **Community Integration Questionnaire (CIQ)**. The CIQ assesses disability for brainy people in three areas: home integration (active participation in the functioning of the home or household), social integration (participation in social activities outside the home), productivity (regular work performance). It is composed of three sub-scores: home integration [0–12], social integration [0–10], productivity [0–7]. The total CIQ score [0–29] is the sum of the 3 sub-scores.

#### Organization of ad hoc data collection

The ad hoc collections at inclusion and throughout patient follow-up will be carried out by trained IRCs. They will produce the compilations necessary for the classification of rehospitalizations in medicine or surgery departments, as well as the research of the lost-to-follow-up. IRCs will be near to participating health facilities to maximize their responsiveness for patient inclusion and ad hoc collection at inclusion.

##### Ad hoc collection of sequelae and elements of pathways

- ◦ **At inclusion (during hospitalization)**

IRCs go to the site at the end of the patient’s stay in order to proceed with inclusion. This collection is done directly with the patient or when the patient is unable to cooperate, with the entourage or the hospital care team. The information collected will be: 1) patient’s postal address at the end of his hospitalization, his telephone number in order to carry out the follow-up visit at 3 months and 1 year after the acute phase as well as the name and address of his attending physician (potentially important information in the search for those lost to follow-up), 2) life pathway before stroke data, 3) administration of three specific assessment scales for different dimensions of sequelae at discharge: NIHSS, BI and mRS.

- ◦ **Follow-up visit at 3 months and 1 year after the acute phase by phone and by mail**

This follow-up at 3 months and 1 year will be carried out by the IRCs, in 2 steps and 2 modes of collection: a mailing and a telephone interview.

- First step: a mail will be sent by the IRC 15 days before the anniversary dates of the 3 months and 1 year after the acute episode. It contains: 1) patient information document about the survey, 2) self-assessment scales exploring different dimensions of sequelae to be completed by the patient (self-questionnaire) or by a caregiver according to the patient’s state of health: FSS, HADS, CIQ-R, 3) explanations about modality of filling in self-questionnaires, 4) pre-stamped envelope to return the self-questionnaires. Patients will have 15 days to complete the questionnaires and return them.
- Second step: telephone interview will be conducted in the week following the anniversary dates of the 3 months and 1 year post-stroke. If the mail questionnaires have not been received, this phone call will serve as a reminder. In case of no answer, the IRC will repeat its call up to 5 times. After 5 attempts, the patient will be considered as lost-to-follow-up.

The information collected will be: 1) life pathway after-stroke data, 2) administration of three specific assessment scales for different dimensions of sequelae: F-TICS-m, Barthel Index and mRS.

##### Classification of hospitalizations during the following year

For all included patients, the re-hospitalizations in medicine or surgery departments that took place during the year of follow-up will be identified from the SNDS base and classified into three categories: 1) readmission for management of recurrence and post-stroke complications; 2) readjustment for post-stroke follow-up and secondary prevention of stroke and 3) re-hospitalizations to manage a clinical situation not related to stroke.

This classification will be carried out in three stages: 1) automated classification based on ICD-10 codes and procedures associated with readmission, 2) for re-hospitalizations not classifiable in an automated way, the IRC will return to the corresponding patient record, within the hospitalization facility and 3) if it is impossible for IRC to classify rehospitalization, a group of experts (including 3 neurologists and a rehabilitation doctor) will meet to classify each remaining hospitalization, based on the information from the hospitalization report.

##### Procedure for searching for lost-of-follow-up

During follow-ups at 3 months and 1 year, if the patient is unreachable, the IRC will contact the treating physician and / or the person around the patient to identify a change in the patient’s place of life or death.

In addition, once all the SNDS links have been completed, the data manager will identify deaths during a hospitalization of unreachable patients during follow-ups at 3 months and 1 year.

### Evaluation criteria

The main evaluation criteria is post-stroke activity limitations by the mRS grouped into three categories ([0; 2]: independence;[3; 5]: dependence; 6: death), at 3 months and 1 year after the acute episode.

The secondary evaluation criteria are:

Other post-stroke sequelae at 3 months and 1 year

- ◦ Post-stroke cognitive disorders by TICS score grouped into 3 categories (24: dementia; [24; 33]: MCI; 34: normal) at 3 months and 1 year after the acute episode.
- ◦ Post-stroke anxiety and depression disorders by HADS score according to 2 sub-score of anxiety and depression, each grouped into 3 categories ([0; 7]: absence of cases; [8; 10]: questionable state; [11; 21]: certain state) at 3 months and 1 year after the acute episode.
- ◦ Post-stroke fatigue by the FSS score grouped into 2 categories (5.5: low fatigue; 5.5: high fatigue) at 3 months and 1 year after the acute episode.

Post-stroke activity limitations by BI grouped into 4 categories (< 20: bedridden; [20–59]: partial autonomy; [60–79]: autonomy for return home; 80: complete autonomy) at 3 months and 1 year after the acute episode. Restrictions on post-stroke participation by the CIQ-R score grouped into 4 categories ([0–12]: home integration; [0–10]: social integration; [0–7]: productivity; [0–6]: electronic social networking) at 3 months and 1 year after the acute episode. Patient clusters responding to pathways with common or similar characteristics (pathway typology).

### Data management and statistical analysis

#### Sample size selection

The primary endpoint is the mRS, measured at 3 months and 1 year after the stroke. Based on data from a pilot study, we assume a prevalence of independence of 51%, dependence of 30% and death of 19%. Several simulations were performed by varying the frequency of exposure to the risk factor studied (very rare to very common). By fixing the risk of first species at 5% and the power of the association test at 80%, to show an odds ratio of at least 2 (odds ratios to move to a higher class: independence to dependence or death, dependence on death), 368 patients should be included to cover a change in exposure to the risk factor studied, ranging from 20% (rare exposure) to 80% (frequent exposure).

The study is stratified on the NIHSS score assessed at inclusion (at the end of the hospital stay for acute episode management) in 3 categories (≤6, 7–16, > 16) and needs to include 368 patients per category of NIHSS or 1104 patients in total (software R v3.3.1).

To account for an estimated 5 to 10% of lost to follow-up, it is necessary to include 1210 patients.

#### Statistical analysis

##### Analysis strategy

The analysis of the assessment criteria will be carried out on available data, i.e. without any replacement of missing data. For the analysis of the primary endpoint, a sensitivity analysis of missing data, using a multiple imputation method, may be considered. The analysis will be carried out using the SAS 9.3 software (or later version) considering a risk of first species fixed at α = 5%. A statistical analysis plan will be defined blind data.

##### Statistical methods

Qualitative variables will be described in terms of numbers, percentages, and 95% confidence intervals according to the exact binomial distribution. Comparisons will be made by the Chi-square, corrected Chi-square or exact Fisher test. A logistic or polytomial regression model will be used to take into account the adjustment variables.

The quantitative variables will be described in terms of mean, standard deviation, median, extent and interquartile range. Comparisons will be made by Student test, Student test for unequal variances, Wilcoxon test, according to the distribution of the variable of interest.

##### Analysis plan

Characteristics of patients at inclusion and during the study.

Patients will be described to the following variables: compliance with eligibility criteria; socio-demographic, clinical, and pathways characteristics. A description of the violations of the protocol, causes of death and abandonment will be made and patients who have died, lost sight of, or abandoned research may be described and compared with other patients.

Analysis of the primary endpoint.

The mRS, the primary endpoint, is an ordered variable with three response modalities. The analysis is based on a Proportional Odds Model, assuming an identical odds ratio for each possible dichotomization of the criterion.

In order to take into account the correlation between the measurement of the mRS at 3 months and that one at 1 year, a random effect on the time will be systematically added to the models.

The odds proportionality hypothesis and the log-linearity hypothesis will be systematically checked.

Analysis of secondary endpoints

- ◦ Typology of the different patient stroke pathways

In order to identify the types of paths, a hierarchical clustering, or latent class clustering, will be carried out.

- ◦ Stroke elements associated with post-stroke sequelae

In order to evaluate the association between the pathway elements and each of the identified sequelae, linear, logistic or polytomic regression models with random effects will be implemented. The assumptions underlying the modelling used will be systematically verified.

- ◦ Social and geographical inequalities in care

The social and geographical characteristics of the patients included will be described and compared, using the descriptive and comparative statistical methods detailed above, between the different types of pathways identified.

### Ethics

All patients will be informed of the objectives of the study and invited to voluntarily participate at the first contact and at the 3 months and 1 year follow-up visit. Patients who will agree to participate will provide oral consent. All information will be kept confidential.

The ObA2 observatory has received the French authority in charge of data protection (CNIL - authorization n°911,201) authorizations necessary for the processing of nominative medical data. Authorizations with the Comité de Protection des Personnes (CPP- number 2018-A01919–46) and CNIL (authorization n° 918,423 (DR 2019–088) have been specifically received for the PAPASépa project.

## Discussion

### Expected outcomes

By integrating a longitudinal dimension and relying on a large cohort, the new knowledge generated by this project will make it possible to identify the barriers and the factors favorable to the outcome of the life pathways, i.e.: 1) information on the elements of the pathway most closely associated with patient functional prognosis, 2) an accurate description of the life pathways, patients with stroke, by defining a typology of these pathways, information currently poorly known, especially on the downstream path, 3) very precise information on the frequency of post-stroke sequelae 3 months and 1 year after the stroke, information currently missing in France.

This information is important for the planning of the offer and thus to the shared and enlightened management of the public policies concerning stroke pathways. Thus, knowledge about the link between the life pathways and functional outcome will bring original information to reduce the impact of stroke by taking into account resources needed meeting the needs of patients and objective information on the inequalities of access to care and on the shortcomings of the healthcare offer.

### Feasibility of the proposed approach

In order to help develop and ensure its feasibility, all the necessary teams and skills are involved in the project: neurologist clinicians, researchers in health services research who know the databases and have the skills in the SNDS data processing, an operational team from the ObA2 cohort, and finally medical informatics teams who will be able to develop data-visualization methods for pathway analysis.

Moreover, the ad hoc collection of sequelae has been tested in a pilot survey and showed its feasibility, its acceptability for patients and caregivers.

Concerning the technical and ethical conditions of the links between the existing databases, respective reconciliations of these databases have already been tested and will be carried out in a secure manner after prior approval by the CNIL (Commission Nationale Informatique et Libertés).

### Justification of methodological choices

#### Study design

Research on pathways must integrate a longitudinal dimension and rely as much as possible on large cohorts. Our project is based on a prospective multicenter cohort (ObA2 cohort), following the life pathway and sequelae of stroke patients for 1 year after the acute episode. Such a design will allow to approaching the place and the role of the temporal and spatial pathway dimensions on the functional prognosis of patients with stroke.

The project relies on data from ad hoc collection and extraction of data from three existing databases. The combination of these sources of information will allow the understanding of the heterogeneity of the life pathway of stroke patients as a whole, in all its dimensions, temporal and spatial, from the acute episode up to 1 year of follow-up.

The cohort will be divided into three strata according to the severity of the patient’s initial clinical condition, as measured by the NIHSS score at hospital discharge. The initial severity of stroke is one of the variables most strongly associated with functional prognosis after stroke and influences the type of life pathway of stroke patients. Thus, it constitutes a potential major confounding factor in the relationship between stroke and sequelae. The NIHSS score at the exit was preferred to the NIHSS at entry because the patient’s pathway is more strongly influenced by the level of hospital dependence at discharge than at entrance, major progress being potentially made during the acute phase of stay.

Other major potential confounding factors, including clinical patient characteristics and elements of the acute phase care pathway, already identified as predictive factors on functional prognosis, are collected and considered in the data analysis (variables related to the patient’s previous condition, variables related to stroke and variables related to the patient’s care pathway prior to hospitalization).

#### Management of the evolving character of the events collected

The three times ad hoc collection and the continuing and prospective collection of pathway events from existing databases will allow the analysis and the management of the evolving nature of the main study issues: sequelae, socioeconomic environment and health events during the year after stroke. This evolutional process is major to describe because it modifies the patient pathway itself. The evolving character of the sequelae throughout the post-hospital journey requires the use of adapted statistical model. This polyptic regression model will analyze the factors associated with sequelae at 1 year, while taking into account the degree of these sequelae at 3 months.

#### Life pathways: perimeter

Despite a collective awareness, as to the inadequacy of the organization of the health system, different realities exist regarding the notion of pathways [38, 44–46].

In the PAPASéPA project, we adopt the global conception of the life pathway of stroke patients. All the elements of care but also the context of life in which the patient evolves (family and social environment, financial aid, income, adaptation of lifestyle and environment, etc.…) will be taken into account.

However, life pathway data are mainly collected during phone interviews using closed-ended questions. Responses may lack of precision and could be not exact. In order to obtain a more precise description of these life pathways access to databases such as the CDAPH (Commission for the rights of disabled persons to independence) would be necessary. This database, which collects all the social benefits people receive, will only be available in the coming years.

#### Types of measured sequelae and tools taken to measure them

The sequelae explored include all impairments (cognitive disorders, anxiety disorders and fatigue), activity limitations (residual and recuperated functional possibilities) and participation restrictions (social consequences) resulting from stroke based on the ICF (International Classification of functioning disability and health). The variables sphincteric disorders and dysphagia will be collected from the Barthel and mRS scales respectively.

While motor impairment, ataxia or aphasia are easily recognized complications, other deficiencies, such as cognitive impairment [47], depression or fatigue are also frequently reported but under evaluated among stroke survivors. These so-called “invisible” deficiencies are thought to contribute to the participation restrictions in daily life activities and impaired quality of life [48]. To address this lack of information, we adopted a multi-dimensional approach to assess the frequency and type of deficiencies with a focus on their daily-life consequences (limitations of activities, participation restrictions) in a cohort of stroke survivors during 1 year after stroke.

The scales measuring each of the dimensions explored are based on elements of feasibility, frequency of use, validation and application condition.

#### Main outcome

The main outcome is the modified Rankin score in three categories. This score gives an overall measure of the level of functional dependence, integrating the ICF components of body function, activity to participation. This is the reference measure found in many publications. Its use in this project will allow national and international comparisons. The defect of reproducibility of its measurement will be taken into account in our project, as following: 1) measure realized by only three people trained according to the good practices of use of the scale before the start of the project, 2) the IRC measuring the mRS during the follow up are not those who included them.

### Representativeness of cohort compared to stroke occurring in the Aquitaine region

A total of 13 health facilities are actively involved in the PAPASéPA project. They include all types of stroke facilities: 5 stroke units, 2 hospitals with neurologist and 6 hospitals without a neurologist. They are spread throughout the Aquitaine region (former Aquitaine region). Their annual stroke patients queue ranges from 70 to more than 1000. In 2016, these 13 centers cared for 5230 stroke patients, which represented 68% of strokes registered in the 2016 regional PMSI Aquitaine database. The comparison between the 2016 ObA2 cohort of these 13 centers and the 2016 PMSI stroke database showed similar age and type characteristics of stroke. Everything suggests that these 13 centers make it possible to constitute a cohort of stroke patients representative of the population of patients treated in Aquitaine for stroke.

### Outlook

Healthcare is changing towards more patient focused care. It’s an international phenomena. The sharing of information systems and the easy sharing of data between professionals is a powerful vector of this evolution, which is conducive to the development of new methodologies for data analysis. The study of the complete patient-centered hospitalization journey in the year following the acute stroke episode requires a longitudinal approach to sequences of health events ordered over time. The reconstitution of these pathways will be based on the chaining of the different hospital stays of the patient, from the first hospitalization during the acute episode of stroke to 1 year of follow-up after the stroke. This project will provide a first overview of the life pathway of stroke patients and have a description of the existing to provide elements in order to propose solutions to the problems posed.

## Data Availability

Not applicable.

## Abbreviations

APA: Allocation Personnalisée d’Autonomie
ARS: Agence Régionale de Santé
CCTIRS: Comité Consultatif sur le Traitement de l’Information en matière de Recherche dans le domaine de la Santé
CDAPH: Comission des droits et de l’autonomie des personnes handicapées
CIF: Classification Internationale du Fonctionnement, du Handicap et de la Santé
CIQ-R: Community Integration Questionnaire-Revised
CNIL: Commission Nationale Informatique et Libertés
EQ5D-3 L: Quality of life EuroQol five Dimension EQ5D-3 L questionnaire
ESD: Early Supported Discharge
FSS: Fatigue Severity Scale
F-TICS: French Telephone Interview for Cognitive Status Modified
HAD: Hospitalisation A Domicile
HADS: Hospital Anxiety and Depression Scale
IADL: Instrumental Activity of daily living
IB: Barthel Index
IDS: Institut des Données de Santé
IRC: Infirmière de recherche Clinique
LHS: London Handicap Scale
MRS: Modified Rankin Scale
NIHSS: National Institutes of Health Stroke Scale
ObA2: Observatoire Aquitain des accidents vasculaires cérébraux
PAPASePA: Parcours des Patients victimes d’accident vasculaire cérébral et Séquelles Post-Accident vasculaire cérébral
PCH: Prestation de Compensation du Handicap
PMSI: Programme de Médicalisation des Systèmes d’Information
QALY: Quality-ajusted life years
SNDS: French National Health Data System (SNIIRAM, PMSI and CépiDC)
SNIIRAM: French National Health Insurance Database
WHO-ICF: World Health Organization’s International Classification of Functioning, Disability and Health
